# Essentials of Cooking

**Published:** 1872-12

**Authors:** J. J. Caldwell, Geo. H. Whaley

**Affiliations:** Late U. S. A., late Surgeon in charge Brooklyn Central Dispensary; Brooklyn, N. Y.


BUFFALO

### Medical and Surgical Journal.

VOL XII. DECEMBER, 1872. No. 5

### Original Communications.

———:o:———

ART. I.—*Essentials of Cooking.* By J. J. CALDWELL, M. D., late U. S. A , late Surgeon in charge Brooklyn Central Dispensary, and Geo. H. WHALEY, M. D., Brooklyn, N Y.

"Cooking almost a divine art." *Johnson.* Regarded from the best view, that is to prolong and cheer our life cooking becomes a science. Hence a truth. Hence divine. 'God sends us meat and the Devil the cooks."—*Rareold Ben.*

Let us see how true this quaint saying may be The learned Liebig, in his researches on the chemistry of food, remarks: "It is obvious that if flesh employed as food is again to become flesh in the body, if it is to retain the power of reproducing itself in its original condition, none of the constituents of raw flesh ought to be withdrawn from it during the preparation of food. If its constitution be altered in any way, if one of the constituents which belongs essentially to its constitution be removed, a corresponding variation must take place in the power of that piece of flesh to be reassumed into the living body. The original form and quality on which its properties in the living organism depend, should be preserved." Hence, boiled meats eaten without the soup, are just so much less adapted for nutrition. Now let us see what is lost both by boiling and too much washing from meats of any kind, viz; soluble phosphate, lactic acid, kreatine and kreatinin, albumen and galatine. The latter is a compound of kreatine and lactic acid, and is readily destroyed by boiling, especially in salt water; this compound being an important stimulus to muscular energy, its loss will readily account for the great debility that follows in cases of scurvy, particularly at sea where the sailor, for any length of time, is confined to boiled salt meat. Indeed, each and every one of these agents has an especial duty to perform in the animal economy; without their presence nothing is left of the meat, save hard masses of fibrine, scarcely digestible: hence, Prof. Liebig has placed the scientific cooking point of meats and vegetables at from two to four degrees below the boiling point, or 212° F. Dr. Letheby, in his lectures on food, makes the following experiment, viz: "Fifteen pounds of meat roasted in the usual way lost 4 lbs. and 4 oz., or over $\frac{1}{4}$ of its weight, and many of its virtues, while with Captain Warren's pot or cooker the loss was not $\frac{1}{2}$ so much, with the virtues all retained. Here, the meat is cooked by its own vapor, the bottom of the pot rests upon boiling water, the meat is placed upon a false bottom—first, that the roasting may go on at less than the boiling point, and secondly, that the gravy may be collected at the deepest point by gravitation. Above this close chamber containing the meat is still another department, connected with the boiling water by pipes conveying steam to this point to cook the vegetables, or whatever may require vapor cooking; the steam first strikes the top of the vessel which acts as a condenser, hence the vegetables too are cooked below the boiling point. Here, also, the same rule holds good as with the meats; the natural qualities are retained within their proper bound, capsules, or cells, while a greater degree of heat would cause them to burst, break forth, and be dissipated or destroyed in a great measure. Again, it is plain, when cooked at this low degree there cannot be any contamination or intermingling of odors, hence, several vegetables or other materials may be cooked in the same compartment without loss of weight, flavor, odor, etc.

Let us contemplate the ingredients that are lost from an excess of heat in the culinary art. First as to albumen, amounting to

60 or 70 parts in a thousand, or about 15 per cent of blood and meats of consumption, ordinarily this component will coagulate at about 150° F. when in a concentrated form, but when diluted and distributed as in flesh it will require fully 212° F. to accomplish the same end; for at and above that point it (the albumen) must expand, lose its watery portions, and become precipitated, hardened, and difficult of solution and digestion. On the contrary, in its natural state it is readily assimilated and carried to the blood directly and rapidly as albumen. The same may be said of the other portions of this material (meats), galatine, and liquor sanguinis, etc., etc., the more completely we get them in their natural state the better.

The soluble phosphates are directly brain and nerve foods, still an excess of heat and evaporation may so alter and change them as to almost lose their identity or cause them to be precipitated and form insoluble compounds only to be thrown off as a foreign matter, thus averting their true interest, (and a fruitful source of rheumatic and other neuralgic troubles.)

The same may be said of lactic acid, kreatine, and kreatinin, they being great stimuli to muscular energy, and found largely in animals of great muscular power.

To any observing mind it is plain, that when all these qualities are maintained, the viand is far more palatable and useful to that great laboratory—the stomach. Thus our bodies are better and more rapidly nourished, our brains more active, our lives prolonged and made happier.

Not the least of the elements that contributed to the unparalleled success of the Germans in their late war, was their magnificent commissariat. Much of man's greatness is due to a happy stomach, and as Tristam Shandy oddly says, "the mind and the body are likened unto a coat and its lining, for you cannot disturb one without rumpling the other," or as the canny Scot expresses it "a fu belly makes a stiff back."

I will here mention, or state, the analysis of liquor sanguinis or meat juices, taking a thousand parts of the liquor, Lehmann says the specific gravity will stand 1028:

| | | | | |
|---|---|---|---|---|
| Water, | 902.90 | 1000 | Fibrine, | 4.05 |
| Solid constituents, | 97.10 | | Albumen, | 78.48 to 90 |
| | | | Fat, | 1.72 |
| | | | Extractive matter, | 3.94 |
| | | | Mineral substances, | 8.55 |
| | | | Water the balance. | |

These are the sum and substance of nutrition and are made directly from the processes of digestion to form new blood and thence new tissues; they all may be, and often are destroyed by just such cooks as the quotation refers to, all, thrown to the winds, so that it often happens at our tables, feasts, frolics, or merry making, that the wayfarer enjoys the real merits of our meats, etc., while the guests gnaw the bones, because the cook owing to ignorance has liberated and scattered these nutrient qualities to the four winds and reserved the husks for the party.

So much for an universal and scientific mode of cooking.

"Grains and roots nourish more than leaves."—*Bacon.*

